# Supplemental Review, and Quarterly Periscope of Practical Medicine, Surgery, &c.

**Published:** 1821-03-01

**Authors:** 


### XVII.

### Supplemental Review,

AND

### QUARTERLY PERISCOPE

OF

### PRACTICAL MEDICINE, SURGERY, &c. &c.

### *WITH COMMENTARIES.*

*Paucis libris immorari et innutriri oportet, si velis aliquid trahere, quod in animo fideliter hæreat.* SENECA.

Duo vitia vitanda sunt in cognitionis et scientiæ studio. ***** Alterum est vitium, quod quidam nimis magnam operam conferunt in res obscuras atque difficiles, easdemque non necessarias. CICERO.

WHEN we cast our eyes around the horizon of medical science and literature, the effusions of thought, observation, and experiment, appear, like the stars in the firmament, or the sand on the shore, without number or end. We find ourselves bewildered with the multiplicity of objects, and are at a loss where to begin, or how to proceed. Could the Father of Physic rise from the tomb to view the products of the toiling press—the endless records of the past—the ever-varying opinions of the present—the vague conjectures respecting the future;—what would be his surprize! He would probably exclaim, in nearly the same language which he held more than two thousand years ago. Alas, my children, it is still the same—"Ars longa et lata—vita brevis et incerta—judicium difficile, et experientia fallax." Seeing then that the limits of our faculties, our time, and even of our paper will not permit us to grasp at much, without running the risk of securing nothing, we shall imitate the humble and industrious BEE—winging our flight of freedom over the flowery fields of literature and science—admiring their beauties, but culling only their sweets.

1. *Aneurisms.** Mr. Pattison is a countryman of our own, a man of ability, brought up in the Glasgow school, and now exercising his talents, like many other of his compatriots, on a foreign soil—if that soil can be called *foreign*, in which the language, the

* Cases of aneurism, with observations. By Granville Sharpe Pattison, Esq. Surgeon of Philadelphia. *Amer. Med. Recorder, April*, 1820.

feeling, the *heart itself* is English, whatever may be said to the contrary by cis-atlantic or trans-atlantic politicians. We have been much gratified by the perusal of these cases and observations of Mr. Pattison, and shall endeavour to present a concentrated analysis of them to the British reader.

We are sorry to be obliged to differ with Mr. Pattison, at the very outset—but it is only on a matter of opinion. He thinks Medical Journals should be records, not of simple, but of *remarkable* experience. Without advocating the practice of filling up journals with common, *uninteresting* matters, we think that the proportion between the common and the extraordinary, *in journals*, should be pretty much the same as in actual occurrences in practice. This, however, is no longer a matter of our concern—we have no reason to complain when the selection is at our own option. In the following sentence of Mr. P's we fully coincide.

"Periodical medical publications are eminently useful. Their expense is moderate, and, if properly conducted, they convey, in a limited compass, not only that which is remarkable in the experience and practice of physicians of one country, but of the world." 194.

We can believe the above, although we have reason to know that its author is just entering on *editorial* functions himself, and consequently is liable to be a little more in love with medical journals than other folks. But to business.

The first case brought forward by Mr. Pattison, is that of a gentleman (Mr. J. M'C.) who died in the 47th year of his age. He was a man of talents and remarkable activity, enjoying excellent health until within six months of his dissolution. In the autumn of 1816, he suffered from what was considered *rheumatic* pains in the lower part of the neck, which did not give way, in the least, to local or general remedies. Attentive examinations of the neck could detect nothing amiss there. The anti-rheumatic plan was therefore persevered in till the patient's death. The gentleman did not desist from business during this period. One evening in April, 1817, he retired to bed in his usual state of health, but was found apoplectic in the morning. In the evening he recovered his sensibility—conversed rationally with his friends for an hour and a half—then relapsed into coma, and expired the next morning.

***Dissection.*** In the head, the cerebral veins were much distended, yet no sanguineous or serous effusion was any where discoverable, although the brain was dissected with the utmost care.

But it was in the chest that the nature of the original disease was at once exposed. A large tumour rose from above the arch of the aorta, projecting sternally, and firmly adherent to the spinal aspect of the sternum.

"Upon separating this connexion, we discovered that the tumour was formed by an aneurism of the arteria innominata, and that the sternum, where pressed on, had become carious. The transverse vein formed by the union of the left subclavian and jugular veins, presented a very uncommon appearance. It had more the character of a ligamentous cord than of a distended vessel; and when opened, it was found filled with coagulable lymph, which completely obliterated its cavity. Being curious to ascertain the cause of this, I traced it carefully downwards towards the right auricle. Upon arriving at the sternal aspect of the aneurismal tumour, the vein terminated that portion of it which crossed the tumour, having from pressure become obliterated. The tumour measured four inches in its transverse diameter, and three in its longitudinal. The depth of the sac from its spinal to its sternal surface was two inches and three quarters. From its situation it completely covered and concealed the trachea and gullet. The whole length of the arteria innominata was involved in it, and those arteries, into which that vessel naturally divides, arose separately, as independent branches from the spinal aspect of the aneurismal sac. Both the superior and inferior thyroidean veins were enlarged and distended with blood; they appeared to be the channels through which the venous blood from the left superior extremity and left side of the head and neck was conveyed to the pulmonic auricle." 196.

We cannot entirely agree with Mr. Pattison that "it is a very rare occurrence for aneurisms of the arch of the aorta to remain unsuspected." These aneurisms are often suspected to exist, when they do not exist; and are not very unfrequently going forward without giving positive indications of their presence. The case of the late Dr. Primrose Blair, related at page 133 of the second volume of the last series, will exemplify this remark, and we could adduce many others bearing on the same point. We agree with our author that it is very remarkable that the pressure of the aneurismal tumour on the trachea and œsophagus, which must have been considerable, as it obliterated the transverse vein, and rendered the sternum carious, caused no symptom indicative of such event during life. The tracheal margin of the tumour projected above the first bone of the sternum, yet the physicians in attendance could not perceive any pulsation in it. Our author thinks, however, that pulsation must have existed. We cannot coincide in opinion with Mr. Pattison, "that the apoplexy which terminated Mr. M'C's life was caused from the obliteration of the transverse vein." We know that aneurismal affections of the heart and great vessels have a remarkable tendency to induce apoplexy, where no obliteration of veins takes place.

Mr. Pattison properly observes, that "whenever local affections are obstinate, we cannot be too particular in examining both local and general symptoms, before we allow ourselves to be satisfied as to the nature of the ailment." We know that pains are often referred to rheumatism, when they are symptomatic of much more serious affections, at a distance from their external seat.

*Case* II. This was a man who had served with the Duke of Wellington through the whole peninsular campaigns, and, of course, had not led a life of ease and idleness—he was, on the contrary, much exposed to dangers and fatigue. Towards the close of the war his health began to fail. The first symptoms of his complaint were pain in both shoulders, particularly in the right. This led the hospital surgeon to treat it as a liver affection, and exhibit mercury, which, of course, did no good. He was therefore discharged the service as an invalid, and returned to his native place, John's-town, in Scotland, and pursued, though in bad health, his original trade, that of weaving.

In the spring of 1818, the pain of the right shoulder almost disappeared, while that in the left became much aggravated, and changed in character—it moved down under the scapula, and became of a throbbing nature. Towards the end of May, a pulsating tumour, the size of a hen's egg, was discovered projecting from under the inferior angle of the scapula. It increased rapidly, and in July it was equal in size to the head of a new-born child, the scapula being tilted forward by it, and the motion of the left upper extremity much impaired. The pain in the situation of the tumour was constant, deep-seated, and severe. The breathing was difficult, and attended with a short disagreeable cough. By the month of November, when our author first saw the case, the tumour had acquired an immense size, and its pulsations were very violent. Emaciation had advanced —the dyspnœa was great, and the patient could only articulate in a low whisper. The pulse was from 80 to 90 at both wrists, and feeble; the radial pulsation not synchronous with the cardiac ones. A consultation was held in Glasgow, where fourteen of the elect of the profession in that city, including Professor Thomson of Edinburgh, examined the case, but they were far from unanimous in their opinions. Of the fourteen gentlemen, twelve thought it either a subclavian or axillary aneurism. Dr. Thomson and Mr. Pattison considered it aortic. Professor ******** ridiculed this idea in the strongest terms possible, and gave a most decided opinion of its being of a different nature.* The patient sunk in six weeks after this consultation.

*Autopsia.* On laying open the chest, the tumour was seen arising from the arch of the aorta, just as it describes the curve, and immediately below where it gives off the left subclavian artery, passing backwards, covering, and completely concealing the bodies of the 2d, 3d, 4th, 5th, 6th, and 7th dorsal vertebræ.

"Upon the right it passed under the pulmonary artery, and reached to within two inches of the inner surfaces of the ribs of that side.

---

* We should not have quoted this passage, were it not to caution and exhort our brethren against the illiberality as well as impolicy of ridiculing each others' opinions in matters of this nature, where we are all so liable to error, and all stand in need of lenity rather than ridicule, in the arduous discharge of professional duties. We have suppressed the name of the individual, because our object is not to hurt the feelings of any one. *Rev.*

On the left the lung was nearly absorbed from its enlargement; that side of the chest being almost filled by the aneurismal tumour. The small flattened portion of the lung which remained betwixt the sternal aspect of the tumour and the ribs adhered firmly to it. When this adhesion was destroyed, we remarked that that portion of the sac from which it had been separated, was formed by the pleura separated from the ribs and thickened. It was opened at this point, and we were enabled by doing so, to expose the dreadful ravages which the disease had committed. The bodies of all those vertebræ pressed on by the aneurism, had become carious, and several of them had been almost completely destroyed. The opening by which the tumour was allowed to protrude through, was formed by the destruction of nearly the whole of the bodies of the 3d, 4th, 5th, 6th, 7th, 8th, 9th, and 10th ribs; a few of the portions of these bones carious were left projecting partially across the passage which communicated betwixt the thoracic and external divisions of the sac. Of the remains of the rest, part had been absorbed, and the remainder were found mixed with the coagula which filled the cavity of the sac. The walls of the cyst towards the thorax were formed by the pleura separated from its natural connexions. Externally, its parieties were composed of condensed cellular substances, strengthened by an internal coating of firm coagulable lymph, which was about half an inch in thickness. Judging from the appearance of the coagulated blood which filled the sac, it seemed to have been nearly all in a fluid state until the death of the patient. When taken out and weighed, it exceeded ten pounds in weight. The external tumour measured on its surface 18 inches in its longitudinal diameter, 14 in its transverse, and 20 in its oblique." 201.

This examination afforded a satisfactory explanation of the symptoms. The pains felt in the right and left shoulders depended, in all probability, on the pressure made on the phrenic nerves. So soon as the circulation made an opening through the parietes of the thorax, the pressure was, in a great measure, taken off the right side. The encroachment of the tumour on the trachea and lungs explains, of course, the dyspnœa from which the unfortunate patient suffered so much. Upon the whole, these two cases are deserving of record among our stock of pathological facts, in order to assist our diagnosis in difficult and anomalous cases.

2. *Erysipelas of the Face, and Inflammation of the Brain.** There are few affections more alarming than erysipelatous inflammation of the face extending to the envelopes of the brain. There is something mysterious about erysipelas, and the practice is still undecided and vacillating. Dr. Dickson, of Clifton, whose professional dis-

* Dr. Dickson, Ed. Journal, No. 69.

crimination is well known, has detailed an instructive case in the last No. of a respected cotemporay Journal, where decisive measures appear to have had a very felicitous result.

The patient, a stout boy of six years, fell into the water, through broken ice, in January 1820, and four or five days afterwards complained of alternate flushes and chills, with pain in the right side of the face, which became red and slightly swelled. Some days of feverishness succeeded, with increase of tumefaction, drowsiness by day, and slight delirium at night. Six days from the commencement of the attack, he was brought to Dr. Dickson, much worse. Six ounces of blood from the arm—a cathartic—febrifuge mixture. On the 8th day he was comatose in the morning, but quite phrenetic in the course of the day. He was bled again to eight ounces, and five leeches to the temples—powders of jalap and calomel. The venesection was ordered to be repeated in the evening, but was neglected. On the tenth day all the symptoms were unrelieved;—rather increased. Blood was decisively drawn until he became faint and quiet, and the proper impression was made on the disease. Nothing more was necessary than keeping up brisk intestinal action by the aid of calomel and other cathartics.

This case, which does credit to the decision of the narrator, shews that when the brain, or other vital organ is inflamed, all other considerations should give place to the indication of checking that process, *coute qui coute*. Nothing but blood-letting, carried the length of arresting the phenomena characteristic of the inflammatory action, can offer any security in such cases.

3. *Disease of the Heart.** We shall introduce this case nearly in the words of the author, omitting only, some unnecessary passages.

" 'W. B. aged eight years, about the 1st of January, 1817, began to complain of indisposition, of restless nights, with some symptoms of nervous affection, and palpitation of the heart.—His indisposition gradually increased, attended with headache and pain through the lower part of the chest. About the 1st of February he was attacked with slight fever, some nausea and distress in the stomach, and increased headache, for which an emetic and some cathartic medicines was administered by the family, and some relief obtained; but the irregular *febrile* affection and indisposition continued, attended with the palpitation of the heart, pain in the head, shoulders, and limbs—he had still kept about and enjoyed a tolerable good appetite. In the early part of March he was violently attacked with general excruciating pain, and high febrile symptoms—the parts mostly affected were the chest, the left shoulder, and the limbs; and in the progress of the disease, was attended with rising of blood, and that followed by expectoration of mucous and bloody matter—he

* Dr. Sherrill, New York Repository, Jan. 1820.

lay about five weeks confined with this attack; attended with such pain and distress in the chest and limbs, that he could not be moved without great difficulty—he gradually recovered after about five weeks, but was still affected with expectoration, erratic pains and palpitation.—At this stage of the disease, I saw him for the first time, in consultation with our venerable friend Dr. Bard—when, from the previous history of the complaint, together with the presence of emaciation, hurried respiration, and fluttering palpitation of the heart, turns of pain through the region of it, also spells of disposition to syncope and nervous irritation, and the apparent enlargement of the *thorax*, we were induced to suggest that there was an organic affection of the heart, or some of its appendages. The prescription which we made was directed rather to palliating the symptoms, than to an expected recovery—he however gradually got better, walked and rode out occasionally; the pain left him by times, and he was entirely relieved of the cough and expectoration. But through the whole progress of the complaint, he was often afflicted with those turns of difficult respiration, some nervous affection, increased palpitation, and great distress, and those spells were brought on or increased by fatigue or agitation of the mind. In the early part of May, he was taken to New-York, in hopes of improving his health, but returned in three weeks not at all amended; he was now more frequently attacked with severe spasmodic affections across the regions of the heart, attended with nausea and vomiting. From this period to that of his death, which occurred on the 25th of September, the same symptoms were frequently renewed with more aggravation, especially with œdematous swellings, great pains, and a gangrene in his feet. He also became so emaciated, that he resembled a skeleton wrapped up in a delicate skin.

" 'The cavities of the thorax and abdomen were laid open; in the abdomen the liver was very much enlarged, and much harder than in a natural healthy state; the left lobe extended itself over the stomach, and reached to the left side, the gall-bladder was very full of thin bile, the spleen was unusually hard, and of a darker hue than natural—the blood vessels of the mesentery were considerably distended with blood and shewed traces of inflammation, the other viscera of the abdomen were in a natural state.

" ' In the thorax, on separating the sternum from its attachments to the diaphragm, it was found to adhere firmly throughout to the lungs and anterior part of the pericardium; those adhesions being dissected, so as to turn up the sternum and cartilaginous portions of the ribs, traces of inflammation and disease were evident in every part of the thorax. The lungs adhered to the pleura, to the mediastinum, to the diaphragm, and were firmly attached to the pericardium; and the attachments were so firm, that the separation had to be made by actual dissection. The substance of the lungs exhibited strong marks of disease and previous inflammation, but were not ulcerated—they were of an irregular, hard, dark texture, but not materially altered from a natural size. On examining the region of the heart, the pericardium appeared to be wanting, or so filled with a large mass that gave a hard spongy feel, that there was no distinguishing the heart from the pericardium, or the one from the other.—By dissecting carefully around this mass, and dividing the great blood vessels, it was taken out altogether, and proved to be the heart enormously enlarged, with the pericardium firmly adhering to it in every part, and so firm was the adhesion, that in separating them, the dissecting knife had to be used for a great part of the process. Having finished this part of the operation, the heart was exposed, and was as large as that of an adult, and of a soft, spongy, yielding, fleshy feel. The ventricles, particularly the right one, were very much enlarged, and the right auricle was of a very uncommon size; the aorta was of a natural size and texture—the pulmonary artery was rather distended and its coats thin—the pulmonary vein was also considerably enlarged. An opening being made into the right auricle and ventricle, a large polypus, of a pale red colour, and spongy feel, was discovered rising from the parieties of the right ventricle, and extending to the left auricle, there enlarging to the size of hen's egg, distending and filling that cavity—a branch or elongation of it passed through the auricle, and ran into the pulmonary vein about two inches; it then divided into two branches, each two or three inches long, running into the branches of the pulmonary vein—at the superior edge of the ventricle at its union with the auricle, a small ossification had taken place—also, in the upper part of the auricle, the part farthest removed from the orifice of the ventricle, there was a second polypus about the size of a small nutmeg, and a small quantity of ossific matter near it.' " 305.

Dr. S. denominates the above a polypus of the heart. We consider it a case of chronic inflammation of the organ, and most probably of that kind connected with a rheumatic diathesis. The polypous concretions, if not *post mortem* formations, were, we apprehend, of very recent origin, and not at all the *cause* of the various symptoms presented in the course of the disease. The case altogether is interesting and instructive.

4. *Neuralgia.** M. Vaidy acknowledges that the *medicina expectans* is not very successful in tic douloureux. We suspect that the same remark might be extended to the Hippocratic treatment of many other diseases; though such a sentiment would be treason in France, where the powers of Nature are considered as almost omnipotent. We too, are convinced of the great extent of Nature's powers—but it is principally her powers of doing mischief, when she ought, and perhaps *means*, to do good. What are the ravages of *disease* (as they are called) but the *erroneous* operations of nature? Will any one suppose that wet applied to the surface can produce abscess in the lungs? No; all, or almost all the disorganizations,

* M. Vaidy, Journ, Comp. Dec. 1820.

discovered after death, result from the ineffectual efforts or re-actions of the system (denominated Nature) to repel the impressions of morbid causes. It is as much then—nay, it is more the duty of the physician to restrain the tumultuous movements of the constitution, than to aid Nature in her sanative processes. It is not improbable, however, that the "*medicina perturbatrix*" may be carried too far here, as the "*medicine expectante*" is on the Continent. And we think it would be much more wise and philosophical for each party, to calmy *examine* the customs of the other, rather than vaunt their own doctrines and practices as the superior ones. The reviewer of Dr. Clark's volume in the last Number of "*La Revue Medicale*," should have made himself acquainted with British medicine, before he pronounced us a faculty of *empirics*. Our Gallic brethren know exceedingly little, speaking generally, of the state of physic in England. They get hold of a few books, usually the very worst, and by these they judge of a whole nation. Thus it was a current opinion in France, that every English physician, on coming to the bed-side of a patient with rheumatism, whether acute or chronic, began to "*pummel him*," "à la Balfour," without paying any attention to constitutional treatment. And so it is, that the eccentricities of an individual are sometimes taken up as characteristics of a whole people. It would be well, perhaps, for the French and British practitioners to *learn* from each other, rather than to *ridicule* or despise each other. Let them be assured that they have *both* a great deal to learn;—and that they have *both* much more reason to deplore their ignorance, than to boast of their knowledge.

*Case* 1. A soldier, 24 years of age, became a patient in the Military Hospital, Paris, for orbito-frontal neuralgia of the right side, coming on every day at 12 o'clock, and lasting four or five hours. It had continued more than a month. It was cured by exhibiting ℥ss. of cinchona before the accession of the paroxysm.

*Case* 2. An orbito-frontal neuralgia, of the remittent kind, was treated, at first, by leeches, but without success. An emetic (three grains of tartrite of antimony) produced copious vomiting and purging; and in six days more the patient was discharged cured.

*Case* 3. Was an old man, of feeble constitution, afflicted with the same kind of neuralgia, returning every day in dreadful paroxysms. Leeches, applied in the line of the nerve, produced only a temporary relief; the cinchona was administered in doses of a drachm four times a day. In three days the disease was arrested; nor was there any relapse six months afterwards.

*Case* 4. In this man the neuralgic affection was in the course of the internal saphena nerve, and regularly increased, in force, for two months. He entered the Military Hospital of Lille, 25th February, 1820. After baths, liniments, &c. had been tried in vain, 30 leeches were applied along the nerve. The blood flowed for 24 hours.

The patient was rendered very weak, but the disease was completely removed. We pass over the sixth case.

*Case* 7. A young man was seized instantaneously with such an excruciating pain in the left leg, along the external cutaneous nerve, that he was obliged to be carried directly to the hospital, during M. Vaidy's visit. The pain was so intense that the soldier cried out with anguish, and implored relief. Thirty leeches were immediately applied. The pain was mitigated by the evening, and next day it totally disappeared.

*The 8th Case* presented orbito-frontal neuralgia, coming on every day at nine o'clock, and lasting five or six hours. Leeches, emetics, the cinchona, all failed to produce relief. The extract of stramonium, in doses of half a grain three or four times in the day, stopped the pain, which did not return till a month afterwards, when it came in the opposite side. The same remedy again removed it. In both instances the stramonium produced only a kind of drunkenness, not particularly disagreeable.

*The 9th Case* was similar to the 7th, being a neuralgia of the leg. Twenty-five leeches removed it.

*The 10th Case*, an orbito-frontal neuralgia was removed in a day or two by the extract of stramonium. In another case of neuralgia, in the line of the biceps muscle, twenty leeches were applied to the arm, with complete success.

M. Vaidy's *12th Case* was an affection of the sciatic nerve, from the hip to the foot, in which a tight bandage, extending the whole length of the lower extremity, removed the complaint.

M. Vaidy concludes that all neuralgiæ depend on an inflammation of the nervous tissues. In this sweeping conclusion we cannot agree. How do his sudden cures by bark, and by compression, tally with this opinion? We do not say that many, or indeed any of the abovementioned cases were what is called real "*tic douloureux*" in this country. They were, however, *neuralgic* affections, of common occurrence, which are often untractable in their nature. That TIC DOULOUREUX is only the severest form of this class, many people believe. We should think, from what we have seen, that the TIC is more generally a sympathetic affection of a nerve, from irritation or disease at a distance, as in the viscera—while the common *neuralgic* affections, of the kind here related, depend, for the most part, on local irritation or inflammation of the nerve itself, or its neurilema. On this account we recommend to our brethren in this country, a trial of the local blood-letting, on the scale practised in France. The application of five or six leeches, in such cases, is not sufficient. The number should be such as completely to drain the vessels of the part, and make a decided impression on the complaint, if possible.

5. *Consuptive Diseases.** In some interesting pathological observations on this class of human afflictions, Dr. Abercrombie first draws our attention to those diseases resembling consumption, and first to sympathetic cough, arising in some unknown way, from irritation seated in some other organ, or part. Nothing can be more certain than the facts, however we may chuse to explain them. Valsalva found the cause of cough in the brain—Lieutand in the frontal sinuses, there being purulent expectoration, without breach of substance in the lungs—De Haen, in the meatus auditorius and uterus. Every one has seen *nervous* cough. The most remarkable one in Dr. A's experience occurred in a stout plethoric young lady, continuing for an hour or more each paroxysm, and nearly strangling the patient. In the intervals, of four or five days, she was in perfect health. The cough was removed by full bleeding from the arm.

Nothing is more common than *stomach* cough. Indeed, when we consider the distribution of the *par vagum*, we cannot wonder at the frequency of respiratory disturbance from gastric irritation. Paroxysms of asthma are every day induced by indigestion, and coughs which resist every other means are frequently cured by laxatives, and especially alterative eccoprotics. In young females, particularly in the higher ranks, a feeling of oppression across the epigastrium, or fixed pain in the side, with paleness, languor, inappetency, insomnium, small frequent pulse, short dry cough, quick respiration on any exertion, alternate flushings and chills, costive bowels, and scanty menses, present a train of symptoms which create apprehension of incipient phthisis. It is most successfully treated by country air, gentle exercise, and a combination of tonics with gentle laxatives—especially sulphate of iron and aloes, two grains of the former, with as much of the latter as will keep the bowels free, thrice a day. Friction of the body is useful, but strong purgatives must be avoided. Warm clothing, the tepid bath, and ultimately the cold bath, complete the cure.

Every one must have seen cough, and uneasy respiration from hepatic affections. In these cases the cough is at first dry, but even this sympathetic irritation of the pulmonary lining membrane, if long continued, will be attended with expectoration of mucus, pus, or even blood. Deranged function or structure, in short, of any abdominal viscus, will occasionally produce cough, and very many of the phenomena of incipient consumption; hence the necessity of minute examination before we decide on the treatment, or venture on prognosis.

Hepatic abscess bursts sometimes through the diaphragm into the lungs. Here mechanical dilaceration adds organic to sympathetic disease, and death is too generally the result.

*Laryngeal Phthisis* is very often confounded with pulmonary consumption, and is a most formidable disease. There is frequent cough with purulent expectoration, sometimes blood, gradual emaciation,

---

* Dr. Abercrombie, Ed. Journal, 66.

hectic fever, and ultimately colliquative diarrhœa, the patient dying with all the symptoms of phthisis. Yet, on dissection, the lungs are found healthy, and ulceration is discovered on the inner surface of the larynx or trachea, sometimes with caries of the cartilages, or irregular fungous elevations of the mucous membrane. We have seen several cases of this melancholy disease. In every one that was carefully examined, the patient complained of some local pain or uneasiness about the larynx, when closely questioned, but not otherwise. Pressure will sometimes develop this pain. The voice is usually affected with hoarseness or huskiness, and is sometimes almost inaudible. The cough is generally in severe paroxysms, so as to excite vomiting sometimes. The laryngeal irritation being communicated to the mucous membrane of the lungs, a copious viscid expectoration from thence, streaked with pus or blood from the ulcer, is thrown up. Hence too the breathing is sometimes affected, or palpitation and other suspicious symptoms of cardiac affection induced. As the disease can only be expected to be arrested during the *first*, or inflammatory stage, anterior to ulceration, it is of the greatest consequence to detect it in its nascent movements; the treatment in such stage is obvious. Bleeding, blistering, antimonials, starvation, purgatives, quietude, and mercury, after the other means have been used, are the principal remedies. After *ulceration* has taken place, nature may, but art, we fear, can seldom, arrest the progress of the malady.

Laryngeal ulceration often occurs in conjunction with common tubercular phthisis—M. Bayle thinks as often as one in six—sometimes as the primary, sometimes as the secondary affection.

Of *chronic inflammation* of the mucous membrane of the lungs imitating, and sometimes producing consumption, we have treated in our last number.

The lungs are not exempt from healthy phlegmonous abscess which, bursting into the bronchiæ or cavity of the pleura, in great quantity may occasion death. Or it may be spat up in pints or quarts for many days, leaving a cavity, like any other abscess, and, like any other abscess, healing kindly, or very protractedly, exhibiting most of the symptoms of pulmonary consumption, and sometimes wearing out the patient with hectic fever.

On hæmoptysis and its consequences Dr. Abercrombie makes many interesting observations; but we doubt whether the different modifications which he has set forth of this alarming accident, can be easily distinguished in practice. The *first* modification is that from turgescence of the pulmonary vessels, whether from obstruction to the blood's return to the left side of the heart, produced by disease of the heart, or induration of the lung itself. This state resembles simple apoplexy, and by the French it has long been termed so. It may be called *acute hæmoptysis*, and requires the same treatment as pneumonia. Under such treatment it will generally do well. The breach of continuity in the lungs resembles that from a cutting instrument, healing by the first intention, or advancing to suppuration and purulent expectoration. In sound lungs this last process may terminate in health—in scrofulous constitutions, it too often ends in consumption.

In the *second* variety, our author thinks the blood comes from the vessels of the bronchial membrane, preceded and attended by less dyspnœa and thoracic oppression, than in the *first* species; but rather with a sharp irritating cough. The blood is in smaller quantity, and mixed with frothy fluid in the early stages—afterwards with bronchial expectoration. It is nearly allied, if not always accompanied by, inflammation of the bronchial membrane, often followed by purulent expectoration and all the phenomena of phthisis, affording, however, a more favourable prognosis. It is, like the *first*, an acute disease.

The *third* modification is *chronic;* apparently resulting from immediate rupture of a vessel, the consequence of disease in its coats. The subjects are pale, feeble, and scrofulous-looking, the pulse being weak and low. It seems connected with tubercular disease. It is apt to terminate in phthisis, or dangerous chronic disease of the mucous membrane.

In the *fourth* variety the vessel gives way from ulceration, in true consumption. It is preceded by purulent expectoration. It is generally fatal; and at best, admits only of palliation.

To these various affections resembling consumption, Dr. A. thinks we might add some remarkable cases in which the symptoms were kept up by extraneous bodies lodging in the bronchia, of which Mr. Howship has published a remarkable case, and Mr. Arnott another.

Dr. A. has offered these observations as a slight and imperfect outline, leaving it to others to fill up the detail. He has industriously collected cases in illustration from various quarters, for which we refer to the excellent paper itself. Dr. A. has laid the profession under great obligations to him for his zealous and able endeavours to advance the science of pathology.

6. *Colchicum** Dr. Williams, in this paper, again calls the attention of the profession to the beneficial effects of the colchicum, in venereal rheumatism, and other painful diseases—shewing the superiority of the seeds over the root of the plant. Dr. W. thinks venereal rheumatism is invariably an asthenic disease, seldom accompanied by pyrexia or hectic—occasionally by eruptions and blotches on the skin, and by nodes—"always by emaciation, and by pains affecting principally the bones and larger joints." Affections of this kind, he thinks, have deprived the army and their country of many brave young men, its brightest ornaments. In three cases of this kind he has found the vinum colchici, of which we gave the formula in our first number, afford not only almost immediate relief, but an early cure. He thinks it necessary, however, to caution the medical prac-

* Dr. Williams. Med. Repos. No. 85. Jan. 1821.

titioner against the too sanguine expectation of success, when the patient is afflicted with acute pain in the bones or their fibrous coverings, *arising from nodes*. For the detail of cases the reader must refer to the journal quoted. The vinum colchici was generaly administered in doses of a fluid drachm, or a drachm and a half, twice a day in water.

In those distressing pains in the back, loins, or chest, arising from deranged actions of the uterus—and in those anomalous pains to which young females are subject, prior to puberty, our author has experienced the most decided testimony of the safety and efficacy of the medicine; but the state of the bowels should be attended to, and fruit, fish, broth, fat meats, eggs, pudding, and pastry avoided, diminishing, by one half, the usual quantity of drink. Dr. Williams thinks the superiority of the seeds over the roots of colchicum proved almost to a demonstration. The *latter* he characterizes as "an untractable, because a most capricious, remedy." In a recent case, he says, the root proved fatal. The seeds, on the contrary, he thinks have the justest pretensions to uniformity of effect.

7. *Hooping Cough.* Dr. Robertson (in the January No. of the Medical Repository) has made some observations on the treatment of this disease, considering it as at first spasmodic, but afterwards inflammatory, he properly recommends as much of the antiphlogistic treatment as may be necessary to guard against the consequences of inflammation in the respiratory apparatus, appeasing the cough by belladonna, rather than cicuta or hyoscyamus—determining to the skin by warm clothing, tepid bath, small doses of antimony, and a gently open state of the bowels. Sudden changes of temperature are to be avoided, although there is seldom any necessity for preventing children, of previously sound constitution, from moderate exercise in the open air. Of all the remedies, however, which our author has had occasion to employ in hooping cough, frictions on the region of the stomach with the tartarized antimonial ointment, have been the most undeviatingly useful. The eruption on the stomach is frequently accompanied by a moderate degree of inflammation about the pudenda, in females, with a slight eruption of minute pimples—on the occurrence of which, the disease, he avers, uniformly begins to abate. He reprobates the indiscriminate exhibition of emetics in hooping cough, as endangering congestion in the brain, and even in the lungs. The object of determining to the surface by emetics may, he thinks, be effected by other and safer means;—and as for evacuating the phlegm or mucus from the lungs, he questions their power in effecting this object. We think our author has erred in his observations on this head. The theory of the action of vomiting is by no means clearly ascertained; but we have seen such decided expectorating effects from emetics, both in hooping cough and other diseases, that we cannot consent to be argued, on theoretical principles, out of the facts which we have witnessed, without any theory on the subject. The preceding observations apply principally to the disease in its early stages. In the chronic state of the disease our author questions the general utility of change of air—and especially the opinion that, if it do no good, it can do no harm. He has seen some lamentable instances to the contrary. He thinks the removal should be but a short distance from home, "and the new abode should be chosen in every thing resembling the former one," avoiding elevated and exposed situations, as well as those that are too low and damp, or within the range of exhalations from stagnant waters or flooded meadows. Inland situations are preferable to the coasts. The advantages of change of air, he thinks, may sometimes be obtained by change of rooms and habits, at home. Upon the whole, we think favourably of this paper, though differing from the author on several points.

8. *Disease of the Heart.** In our last No. p. 458, we detailed a very remarkable case of *apparently* organic disease of the heart, terminating favourably, after a train of symptoms that seemed to dissipate the most distant prospect of recovery. We have here to state another case that, at one time, appeared nearly as formidable as that related by Dr. Gogiran, at Thoulouse. Physicians are too apt to despond, and give unfavourable prognoses when they meet with *symptoms* of organic disease of the heart. But they should recollect that disordered function will assume every symptom, and exhibit every phenomenon of incurable change of structure in the organ of the circulation; and that, in reality, there is not any certain diagnostic by which we can positively decide between the two affections; consequently we are to be guarded in our prognosis, and use means of relief in every instance before we give the case up as hopeless.

Dr. Robbins states that, on the 14th July, Mrs. Smith, a widow, 39 years of age, was visited by him, in a narrow and dirty close, in Edinburgh, where he learnt the following history. In the beginning of January, she was attacked with violent throbbing about the region of the stomach, and also about the heart, increased by any exertion of ascending an acclivity. Her respiration was short and difficult—expectoration of mucus and blood—sense of weight and suffocation at the chest, much increased by the recumbent posture, especially on the right side—no appetite—bowels regular—night sweats—urine natural—œdema of the lower extremities—pain in the right shoulder and arm. No thirst or fever—great weakness and emaciation. These symptoms had continued without abatement till April, when she sent for Dr. A———, who bled her, and pursued a strict antiphlogistic treatment, which considerably relieved her.

About six weeks ago, all the former symptoms returned with unusual violence, as she could not lie down at all, and the little sleep

* New-England Journal, October, 1820.

she has got in the sitting posture, is attended with frightful dreams, and fits of coughing.

"The heart beats with such violence that I can easily count its pulsations at the distance of several rods. Pulse 130, small, hard, quick, and wiry. Her countenance is anxious and sunken—mind gloomy; bowels perfectly regular—night sweats profuse, and debility extreme—dyspnœa and cough violent, but no expectoration of blood—the lower extremities are swelled, and the shoulders and joints painful." 343.

Dr. R. ordered her digitalis and sulphuric acid. July 15th, bled her to 25 ounces; the same medicines. She slept better this night than she had done for a long time. Still "the violent beating of the heart continues to raise the integuments high above the usual level." Pain in shoulder and arms continues, with sense of fulness and heaviness in epigastrio. In a few days she found herself much better, and was able to lie down and get some sleep. On the 19th July, "a pulsating tumour extends high above the right clavicle, which communicates to the finger pressing it a strong thrilling sensation." The same medicines, with a blister to the chest. On the evening of the 21st, she had a fit of vomiting, and was much worse. She confessed she had "ta'n a wee drap o'Highland whiskey." After this she got a little better; but on the 29th July she was all back again.

"Has had constant nausea these two days— pain in the chest, and cough begin to return—pain in the arms exceedingly severe, sharp, and lancinating, so as to prevent her from sleeping, and their soreness extreme; they are considerably swelled, as also are the hands. The beating at the heart is more violent, and palpitation of the tumour above the clavicle more strong than ever. Pulse 84. She has no appetite, and her countenance looks anxious again." 344.

An anodyne medicine was given thrice a day. On the 31st July, she vomited a considerable quantity of blood, succeeded by syncope, which was removed by cordials. After this the symptoms were signally mitigated, while taking the anodyne medicine. Her appetite returned—the pain left the arms—the heart regained its tranquil action—"the palpitation above the clavicle is scarcely perceptible"—pulse 84 and natural—in short, by the 9th of August, she was in as good health as ever she had been in, and continued so.

Our author heads the case as "Organic Disease of the Heart," and closes it with these words:—"The disease which I have related, was an effusion of a watery fluid between the heart and pericardium, succeeding inflammation of those organs." We are very far from believing the disease to have been either an *organic* affection of the heart, or hydro-pericardium. Had it been either of these, Dr. Robbins would not have restored her to perfect health in *twenty-five days*. We conceive (for we would not be so positive as our author) that the disease was a pulmonary affection, relieved by the vomiting of blood; and that the cardiac symptoms were symptomatic or sympathetic.

9. *Ruptured Tendo Achillis.** Personal experience is far superior to all other kinds. Dr. Edmonston ruptured the tendo achillis in dancing. A week, passed under bandage and rest, produced little amendment, especially when the least motion was attempted. Dr. E. now shaved the leg, and applied adhesive straps along the whole of it, from the ancle to the knee, making them overlap by several inches, and causing them to produce equal pressure. Over all was put a tight elastic worsted stocking. In three days after the application of the straps, he could walk with the aid of a stick, dragging, of course, the injured leg after the other. He continued to walk two weeks longer in this guarded manner, and soon recovered. Dr. E. thinks, and probably with justice, that, in all sprains, some fibres are either actually ruptured, overstretched, or so bruised as not to be able to contract or relax as in health. In such cases, he has employed adhesive straps, so as to give a uniform support, with much advantage. We think the suggestion an ingenious one, and likely to be practically useful.

10. *Stomach affected by cold water.*† There is strong reason to believe that the stomach may be seriously injured by gelid potations, even where there is no preternatural exhaustion of the system, or perspiration on the skin. The following case is illustrative of this supposition.

"S. M. G.,—the subject of these remarks, is a young man of about 21 years of age, of very delicate constitution, and of a pulmonary predisposition. He is by occupation a store-keeper, and has been in the habit of walking to and from Philadelphia (a distance of about 16 miles) in warm weather, generally once a week, and had performed one of these journeys but two days previous to his attack. On the 26th of June, at about twelve o'clock, his father perceived him on the shop counter, in a reclining position, as though he were asleep, but of so pallid a hue as to induce the belief that he had fainted. On examination, he was found to be in a state of complete syncope; and when I saw him (not fifteen minutes afterwards) he was without pulse, his wrists cold, and in short, to all appearance, lifeless. I began immediately to use external stimulating applications of various kinds, such as sinapisms, water, almost boiling hot, to the extremities, frictions with hartshorn, spirits of turpentine, &c. For half an hour, there was scarcely any pulsation perceptible; the respiration was at long intervals and laborious, and the jaws were so locked, as to render it almost impracticable to introduce any thing into the stomach. During this time, no cause could be assigned for the attack, as he had not been fifty yards from

* Dr. Edmonston, Ed. Journal, 66.

† Dr. Mitchel, New York Repository, Jan. 1820.

the store, nor engaged in any sort of fatiguing employment. Presently, however, he was seized with violent spasms of the stomach, which continued for two or three hours; to relieve which, I gave a tea-spoonful of laudanum every fifteen or twenty minutes. I now suggested the probability that my patient had been drinking freely of cold water, which, acting on his empty stomach, had produced all the unhappy symptoms. I found that the accident occurred just before the usual dining hour of the family, and that my patient had eaten nothing since his breakfast; but no one knew any thing relative to the cold water, nor were any of the family disposed to believe that a person could be injured by cold water, unless previously heated. Caring but little, however, for the cause of the attack, I persevered in the use of the external and internal stimulants, until twelve bottles of mustard, and three of Cayenne pepper, were consumed, together with a quantity of other articles. At about six o'clock p. m. and not until then, he spoke a few words, but soon relapsed into a state of stupidity, the spasms of the stomach having been nearly removed. The treatment was continued as rigorously as before, until nine o'clock at night, when he again spoke, and held a conversation of some length. During this conversation, he was interrogated relative to the cold water; and he assured me, that the last thing he could remember, previous to his attack, was, that he took a free draught of cold water, from a particular pump in the borough, which he specified, well known as the coldest water in the neighbourhood." 810.

11. *Ligature of the common Iliac Artery.** Accidents or diseases requiring the ligature of the second largest vessel in the human body will not often occur. Yet they may happen again, as they have already happened, and, consequently, it is proper to place on record the following case, the detail of which, however, we shall considerably abridge.

During the riots of 1812 in Baltimore, a labouring man, 38 years of age, received a musket shot, which entered the left side of the abdomen, passed through the intestines, and lodged in the sacrum. Dr. Gibson was on the spot. A gush of blood came from the wound. The man being of a thin habit, Dr. G. by thrusting the fore finger of his left hand into the wound, reached the orifice of the bleeding iliac artery, and stemmed the hæmorrhage. The moment he removed his finger the bleeding returned. He, therefore, kept the wounded artery pressed, till Drs. Owen, Hall, and several other medical gentlemen, came to his assistance. He determined on attempting the ligature of the artery. We deem it proper to give the steps of the operation in Dr. Gibson's own words.

---

* William Gibson, M. D. Professor of Surgery in the University of Pensylvania. *Med. Recorder*, *April* 1820.

"Still continuing my finger within the wound and pressed upon the artery, I commenced the operation (in the presence of Colonel Mitchell of the United States army, Drs. Owen, Hall, and several other medical gentlemen), by dividing the integuments and muscles of the abdomen, above and below the wound, to the extent of seven inches. The peritoneum was next cut through, the intestines turned to one side until the artery was exposed and slightly detached from the parts in its immediate vicinity. I now attempted to pass the common aneurismal needle armed with a large ligature, under the artery; but, owing to the want of flexibility in the instrument, and the depth of the cavity, I found it impossible to secure the vessel.* I then requested Dr. Hall to pass the ligature through the eye of a common flexible silver probe, curved to rather more than a semicircle, which being conveyed under the artery, the ligature was applied, but not without some difficulty. I now found, that I had tied the left iliaca communis artery, and the pulsation above the ligature could be distinctly perceived, although the circulation, in the upper and lower extremities, was very feeble. I waited for some minutes, to see whether the blood would flow from the lower portion of the artery, but no hæmorrhage taking place, I turned my attention to the intestines, which were wounded in two convolutions. I placed a small thread upon each opening, and drew it with moderate firmness, so as to close up the wounds and prevent the effusion of fecal matter into the abdomen.† The extremity of each thread was cut off near to the intestine, the other was brought forward and secured at the external wound. The large ligature which embraced the artery,

---

* "If I had had the simple but ingenious instrument invented by my friend, Dr. Physick, twenty years ago, viz. a forceps, holding in its extremities a blunt curved needle, with an eye through which the ligature is passed, I should have experienced no difficulty whatever, in taking up the artery. For an account and drawing of this instrument, see Dr. Dorsey's valuable work, the Elements of Surgery."

† "I preferred including the openings of the gut with ligatures instead of sutures, knowing that I could easily cut them away, if any urgent symptoms should follow their application. I was likewise induced to make the experiment, from knowing that Mr. Astley Cooper, and some others, had drawn together apertures in sound portions of intestines by ligatures, after the operation for strangulated hernia, with success; and that experiments on animals had been made with similar results. It is proper to mention in this place, however, that a ligature applied to a piece of intestine, in the manner described, has often been productive of the worst effects. Dr. Physick tried the experiment many years ago; but a violent colic pain, which immediately supervened, obliged him to cut away the threads. On the other hand, I may state, that in a patient of Dr. Martin, of Baltimore, upon whom I operated about three years ago, for strangulated inguinal hernia, and in whose intestine a rent was found of considerable size above the stricture, the ligature was tied, so as to draw the edges closely together, and the ends of the thread were cut off near to the knot. No inconvenience followed the operation, and the patient recovered in the usual time."

was likewise fastened externally, one end being previously divided near the knot. The clotted blood which was found in the neighbourhood of the wounded vessel, and in the cavity of the abdomen, in considerable quantity, was carefully removed; and upon doing this, a slight hæmorrhage took place from the lower portion of the common iliac. The bent probe was again carried beneath the vessel, and the ligature drawn so as to stop the bleeding. The parts were now permitted to close up, and to occupy their former situation; and the edges of the external wound were kept in contact with each other by adhesive straps, and a slight dressing. The operation was severe and protracted, and the patient reduced exceedingly low. A pupil watched him during the night, and kept the limb continually covered with warm flannel, and bladders filled with warm water applied to the foot." 187.

*2d day.* The patient passed a restless night, having had spasmodic twitchings of the left thigh and leg. Pulse at the wrist fuller and stronger— abdomen not swollen or tender—left lower extremity warm.

*3d day.* Patient still better—passed urine and fæces freely—freer from spasms, probably in consequence of having taken 15 drops of laudanum every three hours—countenance somewhat flushed—pulse encreased in frequency—pain on pressure of the abdomen. "The warmth of the thigh has increased, but the knee, calf of the leg, and foot are cold, and comparatively insensible."

*4th day.* Patient passed an uncomfortable night, notwithstanding the laudanum. Complains of intense headache—tongue furred—great thirst—gastric irritability—inappetency—urine highly coloured, fæces very fetid—pulse 140. The temperature of the thigh has not encreased much, but the leg is warmer. Complains much of the sole of the foot—abdomen more swelled and painful than yesterday—considerable thin offensive discharge from under the dressings. The dressings being removed, the wound gapes, and shews no sign of granulation. A large blister to the belly.

*5th day.* Patient extremely restless and delirious—pulse very quick and irregular—heat of the whole limb materially increased—wound not improved in appearance—matter more offensive—swelling of the abdomen undiminished.

*6th day.* Patient feels relieved, and passed a good night—thirst diminished—urine not so high-coloured—"heat of the left thigh greater than that of the right, but the foot yet remains very cold." Abdominal tumefaction the same—discharge from the wound copious, and mixed with large clots of blood—pulse 130—patient very weak.

*7th day.* Little variation in the general symptoms.—"The limb appears to be well supplied with blood, and the heat of the foot is nearly equal to that of the opposite side."

*8th day.* Edges of the wound sloughy, with some appearance of adhesion at certain points. The discharge is mostly of an offensive kind, and mixed with blood. The heat of the thigh and leg is still greater than that of the opposite side.

*9th day.* A tremendous hæmorrhage. On removing the dressings and coagula, the bottom of the wound could be reached by the finger—the vessel was felt distinctly pulsating above the upper ligature but no blood flowed.

*10th day.* A gush of blood at 10 o'clock to day, which soon stopped.

*11th day.* Symptoms all of the worst order; but he lingered out with occasional hæmorrhages, and all the phenomena of abdominal inflammation, till the 15th day, when he expired.

" *Dissection.*—On opening the abdomen a few hours after death, a very large quantity of air was extricated; and a dark brown fetid matter, mixed with blood, issued from the wound, in the neighbourhood of which it was extensively diffused. The intestines were glued to each other in every direction, bearing evident marks of violent preceding inflammation. A small opening was discovered in the intestine, from which the ligature had separated; but an adjoining convolution had adhered to it so closely, as nearly to block up the cavity. The other ligature retained its situation, being loosely connected to the everted edges of the wounded gut, and surrounded, for some distance, by coagulable lymph. The peritoneum adhered to the sides of the wound, and in several spots to the surface of the intestines. It appeared to have suffered, more or less throughout, from inflammation. No fecal matter could be found effused into the cavity of the abdomen.

" Upon inspecting the vessels of the abdomen, I found that I had placed two ligatures upon the *common iliac* artery of the left side; one about half an inch below the bifurcation of the aorta, and the other immediately above the division of the artery into the external and internal iliacs. The upper ligature was found detached from the vessel, which projected and preserved the circle of its mouth entire. Its cavity, as well as that of the aorta, for a short distance, was blocked up by a recent coagulum of blood. The coats of the artery at its extremity had retained the impression of the ligature, and were of a pale blue colour throughout the margin of the circle. *No union whatever had taken place.* The extremity of the lower portion of the iliaca communis, was still embraced by the ligature; but on dividing this with the knife, and splitting the cavity of the vessel, I found that a partial adhesion only had been produced, and there was no vestige of a coagulum. The blood had injected the cellular membrane in the vicinity of all the large vessels, and had almost separated the iliaca communis and lower part of the aorta from their connexions. The bullet was found imbedded in the upper and left side of the sacrum. Having proceeded thus far, I was interrupted by a jury of Inquest, assembled to examine the body. I had made arrangements to inject the limb, in order to ascertain the precise manner the blood had found its way to the lower extremity; but the friends of the patient would not listen to the proposal, and the times were too tumultuous to attempt to accomplish my purpose without their approbation." 191.

Our surgical readers will readily grant, that little hope could have been entertained of success in the above case, from the beginning. Yet it is surely important to know that, the common iliac trunk may be tied without cutting off permanently, the supply of blood to the lower extremity, since the operation may possibly become necessary in cases of aneurism. It is on this account that we have laid a full analysis of the paper before the British Public.

---

